# The Ros/MucR Zinc-Finger Protein Family in Bacteria: Structure and Functions

**DOI:** 10.3390/ijms232415536

**Published:** 2022-12-08

**Authors:** Monika Janczarek

**Affiliations:** Department of Industrial and Environmental Microbiology, Institute of Biological Sciences, Faculty of Biology and Biotechnology, Maria Curie-Skłodowska University, 19 Akademicka, 20-033 Lublin, Poland; monika.janczarek@mail.umcs.pl

**Keywords:** Ros/MucR family, bacterial zinc-finger domain, metal-binding proteins, symbiotic bacteria, pathogenic bacteria, DNA-binding domain, H-NS proteins, DNA bridgers

## Abstract

Ros/MucR is a widespread family of bacterial zinc-finger-containing proteins that integrate multiple functions, such as symbiosis, virulence, transcription regulation, motility, production of surface components, and various other physiological processes in cells. This regulatory protein family is conserved in bacteria and is characterized by its zinc-finger motif, which has been proposed as the ancestral domain from which the eukaryotic C_2_H_2_ zinc-finger structure has evolved. The first prokaryotic zinc-finger domain found in the transcription regulator Ros was identified in *Agrobacterium tumefaciens*. In the past decades, a large body of evidence revealed Ros/MucR as pleiotropic transcriptional regulators that mainly act as repressors through oligomerization and binding to AT-rich target promoters. The N-terminal domain and the zinc-finger-bearing C-terminal region of these regulatory proteins are engaged in oligomerization and DNA binding, respectively. These properties of the Ros/MucR proteins are similar to those of xenogeneic silencers, such as H-NS, MvaT, and Lsr2, which are mainly found in other lineages. In fact, a novel functional model recently proposed for this protein family suggests that they act as H-NS-‘like’ gene silencers. The prokaryotic zinc-finger domain exhibits interesting structural and functional features that are different from that of its eukaryotic counterpart (a βββα topology), as it folds in a significantly larger zinc-binding globular domain (a βββαα topology). Phylogenetic analysis of Ros/MucR homologs suggests an ancestral origin of this type of protein in α-*Proteobacteria*. Furthermore, multiple duplications and lateral gene transfer events contributing to the diversity and phyletic distribution of these regulatory proteins were found in bacterial genomes.

## 1. Introduction

The Ros/MucR family encompasses prokaryotic zinc-finger (ZF) proteins that interact with DNA and regulate the transcription of genes required for both symbiotic and virulent interactions between bacteria and their respective hosts [1,2,3,4,5,6]. Among the members of this family, there are proteins from different bacteria (mostly α-*Proteobacteria*) colonizing various ecological niches, including symbionts and pathogens of plants and mammals. Very often, the bacteria in this group live within or in close association with the cells of their host, and these interactions with the eukaryotic host cell are essential for the life of these bacteria. Due to the close phylogenetic relatedness, these organisms use common genes and strategies facilitating their interactions with their host, and the gene encoding regulatory proteins Ros/MucR is one of the genes conserved in α-*Proteobacteria*, which is important for host–bacterium interactions. 

In eukaryotic organisms, the abundant ZF domain involved in relevant protein–nucleic acid and protein–protein interactions consists of up to 30 amino acids (aa) and a zinc ion tetrahedrally coordinated by two histidine nitrogens and two cysteine sulfurs [7,8]. In contrast to Eukaryotes, the first prokaryotic ZF domain was identified by Chou and others in 1998 in a protein from *Agrobacterium tumefaciens* named Ros [9]. Since then, Ros homologs have been identified and characterized in many other (both symbiotic and pathogenic) bacteria, all bearing some interesting new features representing some differences in comparison to the eukaryotic domain. In contrast to the eukaryotic classical ZF domain (also called C_2_H_2_), which represents the most common class and forms a compact ββα architecture consisting of a β-sheet and an α-helix, ZF in bacteria shows several structural and functional differences [10]. The pleiotropic effects of mutations in genes encoding Ros/MucR proteins confirm the importance of these proteins in the functioning of bacterial cells. 

From an evolutionary standpoint, the prokaryotic ZF has been proposed as the ancestral domain from which the eukaryotic C_2_H_2_ ZF domain has evolved [11]. The structural and functional similarities and differences between the prokaryotic and eukaryotic ZF have been largely documented [7,8]. The two domains are similar in the tetrahedral coordination of the structural zinc ion and in the existence of the surrounding ββα topology, but they differ in the presence of an additional α-helix and a larger hydrophobic core in the prokaryotic ZF. In this review, recent data concerning the structural characterization and biological functions of regulatory proteins from the Ros/MucR family have been compiled, and the possible roles of these regulators in both genome evolution and bacterial adaptation have been discussed.

## 2. Pathogenic Bacteria

### 2.1. Agrobacterium Tumefaciens

**Figure 1:**
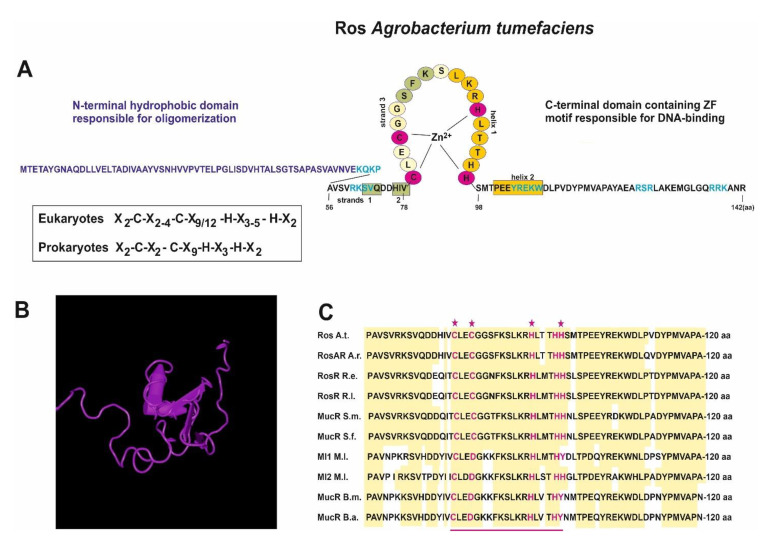
(**A**) Schematic representation of the amino acid sequence of *A. tumefaciens* Ros, including C_2_H_2_ ZF motif. Amino acids engaged in binding of Zn^2+^ ion, forming α-helixes and β-strands are marked in purple, orange, and green, respectively. Amino acids located in hydrophobic regions of Ros are marked in blue color. (**B**) Three-dimensional structure of Ros (PDB database, 2JSP); (**C**) Comparison of C_2_H_2_ ZF motif sequences of different MucR/Ros representatives. Cysteines and histidines involved in Zn^2+^ binding are marked in purple asterixes, and identical amino acids are shadowed in light yellow, respectively. Abbreviations: A.t.—*Agrobacterium tumefaciens*; A.r.—*Agrobacterium radiobacter*; R.e.—*Rhizobium etli*; R.l.—*Rhizobium leguminosarum*; S.m.—*Sinorhizobium meliloti*; S.f.—*Sinorhizobium fredii*; M.l.—*Mesorhizobium loti*; B.m.—*Brucella melitensis*; B.a.—*Brucella abortus*.

The first prokaryotic C_2_H_2_ ZF domain was identified in the transcriptional regulator Ros from *A. tumefaciens*, suggesting that this type of domain, originally thought to be confined to the eukaryotic kingdom, may be more widespread, including also the prokaryotic kingdom. *A. tumefaciens* is a Gram-negative soil bacterium that is able to cause crown gall tumors at wound sites in dicotyledonous plants. During the infection, this bacterium can transfer a fragment of its Ti plasmid to plant cells. Ros was found to be involved in the horizontal transfer of genes from *A. tumefaciens* to the infected host [12]. Ros is encoded by a chromosomal gene called *ros* (*r*ough *o*uter *s*urface). This protein functions as a repressor targeting a sequence located in the promoters of *virC* and *virD* operons, the products of which are involved in processing the oncogene-bearing T-DNA region of the Ti plasmid from the horizontal gene transfer from *A. tumefaciens* to plant cells (Figure 1A–C) [1]. 

**Figure 2:**
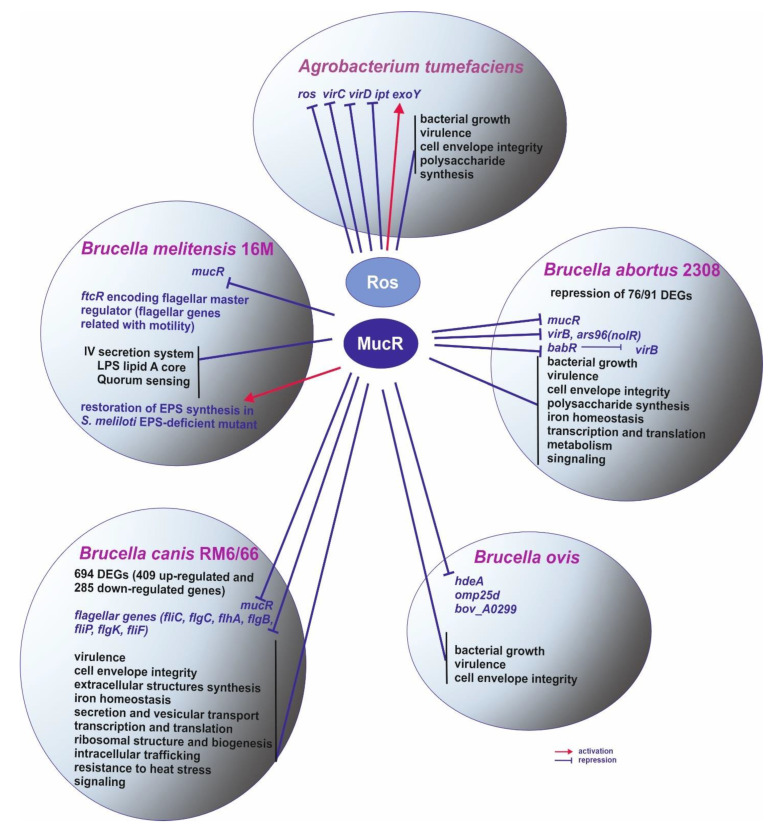
MucR/Ros proteins from different pathogenic bacteria: *Brucella* and *Agrobacterium tumefaciens* and their roles in regulation of expression of various genes involved in several cellular processes and adaptation to environmental stresses.

The Ros-binding site in the promoters of *virC* and *virD* operons has been identified and characterized [13]. It was a 40-bp long A/T-rich sequence motif containing a 9-bp inverted repeat TATATTTCA/TGTAATATA, designated the “*ros*-box”, suggesting that Ros binds as a dimer. The *ros*-box motif was also found in the *ros* promoter region. The Ros protein proved to bind to these regulatory motifs located in the promoter regions of *virC* and *virD* operons and its own *ros* gene promoter [12,14,15]. Although Ros did not appear to affect virulence *per se*, its absence increased the appearance of T-DNA intermediates in *A. tumefaciens* as a result of the de-repression of *virC* and *virD* operons [16]. Ros also regulates the expression of the *ipt* oncogene located in the T-DNA region, which is recognized in plants by transcriptional machinery and encodes isopentenyl transferase required for the synthesis of cytokinins (Figure 2) [9]. Thus, mutations in the *ros* gene resulted in the formation of non-mucoid colonies of *A. tumefaciens* on agar plates and up-regulation of the *virC* and *virD* operons in the absence of induction by plant phenolic compounds. This led to the appearance of T-DNA intermediates in *A. tumefaciens* cells and the de-repression of *ipt*, which activated the production of cytokinin in the bacterial cells [14,16]. 

In *Agrobacterium radiobacter*, a bacterium closely related to *A. tumefaciens*, a homolog of *ros*, i.e., *rosAR*, was found to be required for the expression of *exoY* encoding a glycosyltransferase involved in the first step of the exopolysaccharide (EPS) synthesis (thus being engaged in positive regulation of EPS synthesis) [17].

The *A. tumefaciens ros* gene is highly conserved in members of the *Rhizobiaceae* family (Table 1). It encompasses a single 426-bp long open reading frame (ORF), which encodes a 142 amino-acid (aa) protein. Therefore, Ros is a relatively small protein (15.5 kDa) with a pI of 7.1. The N-terminus of this protein is negatively charged and contains more hydrophobic aa residues than the positively charged C-terminus, which contains a C_2_H_2_-type ZF motif with a sequence C-X_2-_C-X_3-_F-X_2-_L-X_2_-H-X_3_-H-H (i.e., 79–97 aa of the full-length protein) [14,15,18]. However, this C_2_H_2_ ZF domain in Ros differs from the classical C_2_H_2_ ZF in eukaryotic proteins; namely, there are three histidine residues (H92, H96, and H97) and a shorter peptide loop (i.e., a 9-aa spacer) between the first cysteine and histidine residues in the motif (whereas the eukaryotic ZF domain has only two H residues and a 12-aa spacer) (Figure 1A–C). 

In 2006, Esposito and others [19] reported the first complete functional characterization of the DNA-binding domain occurring in the putative C_2_H_2_ ZF-containing prokaryotic transcriptional regulator Ros from *A. tumefaciens.* They confirmed that two cysteines (C79 and C82) and two histidines (H92 and H97) in the Ros ZF domain are engaged in the coordination of the Zn(II) ion in a tetrahedral fashion. Basic amino acids flanking this C_2_H_2_ motif proved to be important in stabilizing the DNA binding, as in eukaryotic ZF motifs [19]. Interestingly, single mutations of either H96 or H97 do not alter the zinc ion and the DNA-binding capability of the Ros protein, whereas a mutation in both residues abolishes the DNA-binding activity and strongly reduces the zinc coordination capability (Figure 1A). This indicated that both these residues function as the fourth coordinating position alternatively. However, it was observed that when H97 was mutated, H96 was able to occupy the fourth position of the zinc coordination, changing its tautomeric form (for details, see paper [19]). 

Interestingly, a deletion mutant of Ros lacking 55 aa at its N-terminus was still capable of DNA binding [19]. In 2007, Malgieri and colleagues [20] described the NMR solution structure of the DNA-binding domain of this protein (called Ros87, region 56–142 aa). The data indicated that this C_2_H_2_ ZF sequence is indeed a part of a significantly larger zinc-binding globular domain possessing a novel protein fold, which is highly different from the fold reported for the classical eukaryotic ZF [20]. The Ros87 globular domain consisting of 58 aa is arranged in a βββαα topology and is stabilized by an extensive 15-residue hydrophobic core. This domain is uniformly rigid and flanked by two flexible tails. Two cysteines (C79 and C82) from the Ros ZF domain are located on the β-hairpin, whereas two histidines (H92 and H97) are located in the middle and at the C-terminus of the α-helix [10,20]. 

Further studies conducted by this research group [11] focused on the evolution of the classical ZF domains with the goal of determining whether eukaryotic ZFs have evolved from prokaryotic Ros-like proteins. Based on computational and experimental data, Palmieri and colleagues [21] indicated that a single insertion of three amino acids in the short loop that separates the β-sheet from the α-helix of *A. tumefaciens* Ros was sufficient to stimulate a structural transition from a Ros-like to a eukaryotic-ZF-like structure. This observation provided evidence for a structurally plausible and parsimonious scenario of fold evolution, giving a structural basis to the hypothesis of its horizontal gene transfer from bacteria to eukaryotes [21].

Moreover, a novel functional model for the Ros/MucR family of proteins, to which *A. tumefaciens* Ros belongs, suggesting that they may act as H-NS-‘like’ gene silencers, was proposed by Baglivo and others [6,22]. Indeed, *A. tumefaciens* Ros homologs Ml1, Ml2 from *M. loti*, and MucR from *B. abortus* show interesting structural analogies with the Histone-like Nucleoid-Structuring (H-NS) protein, which plays a role in the formation of the nucleoid structure [23]. H-NS consists of two functional domains separated by a flexible linker: The N-terminal domain is responsible for the formation of high-order structures that bind DNA via the C-terminal domain. The two domains present in the Ros/MucR family members may function as independent units with specific functions (i.e., the C-terminal domain harboring ZF adopts a conformation that is prone to DNA-binding, regardless of the presence/orientation/interaction of the N-terminal domain, whereas the N-domain itself may control the oligomerization mechanism). Indeed, the N-terminal region (1–55 aa) of the Ros homologs Mls and MucR appear to be responsible for the formation of functional oligomers. This region was not analyzed for many years because of the serious problems with the solubility of the full-length proteins belonging to the Ros/MucR family. Recently, structural characterization of the N-terminal domain of the full-length Ros homologs, Mls, and MucR was provided by Baglivo and others [6,22]. The authors demonstrated that the oligomerization of this protein is a key feature for its proper regulatory function. Moreover, they proposed that prokaryotic ZF proteins belonging to the Ros/MucR family work as H-NS-‘like’ gene silencers by binding low consensus A/T-rich regions in DNA rather than functioning like their eukaryotic counterparts that mainly act as DNA sequence-specific transcriptional regulators [23].

Thus, all recent results support and integrate previous findings about *A. tumefaciens* Ros and its homologs, which suggest that the prokaryotic ZF proteins control gene expression by adopting a mechanism similar to that used by the H-NS proteins found in several other Gram-negative bacteria instead of working similarly to classical eukaryotic transcription factors [24]. 

Furthermore, the prokaryotic ZF (as recently shown for Ros87 from *A. tumefaciens*), which has a bigger βββαα domain with a larger hydrophobic core with respect to its eukaryotic counterpart, represents a valuable model protein to study metal ion interactions with metallo-proteins [25]. The recent data have demonstrated that the DNA-binding domain of *A. tumefaciens* Ros structurally tolerates the Ni(II) ion, albeit with important structural perturbations, but not Pb(II) and Hg(II), and this protein proved to be functional in vitro when the Zn(II) ion was replaced by Cd(II). It was shown that the substitution of the native ion resulted in completely different folding scenarios. These results outline the complex cross-correlation between the protein–metal ion equilibrium and the folding mechanism, proposing this interplay as a key factor in the proper metal ion selection by a specific metallo-protein [25]. 

### 2.2. Brucella spp.

Interestingly, although bacteria of the genus *Brucella* are animal pathogens, they are very closely related to plant nitrogen-fixing symbionts, commonly called rhizobia, including *Sinorhizobium meliloti*, a model bacterium for studying symbiotic plant-microbe interactions. *Brucella* spp. are facultative intracellular parasites causing brucellosis, a chronic widespread zoonotic disease affecting a broad range of mammals, including livestock and humans [26,27,28]. *Brucella* spp. and *S. meliloti* have many physiological similarities. They belong to α-*Proteobacteria* and share not only the intracellular lifestyle in their respective hosts but also a crucial requirement for cell envelope components and their precise regulation for successful infection. Most of the characterized virulence determinants of *Brucella* spp. are associated with their surface and its structural components (i.e., an envelope, a type IV secretion system, and a flagellum). In 2006, *mucR* identified in *Brucella melitensis* strain 16M proved to be an orthologue of *S. meliloti mucR* (Figure 2) [29]. This gene also encodes a ZF regulatory protein and is involved in infection since a *mucR* mutant showed decreased virulence in both cellular and mouse models of infection. A protein encoded by *mucR* affected the surface properties and resistance of *B. melitensis* to various environmental conditions (i.e., oxidative and saline stresses and detergents). This protein also repressed its own transcription and expression of flagellar genes via the flagellar master regulatory gene *ftcR* and affected various cellular processes, such as quorum sensing, the synthesis of LPS lipid A core, and the functioning of the IV secretion system [30,31]. Furthermore, *B. melitensis mucR* was able to restore the synthesis of exopolysaccharide (named EPS-I) in an *S. meliloti* EPS-deficient *mucR* mutant, confirming that this gene is a functional orthologue of *S. meliloti mucR* (Figure 2). Homologs of *B. melitensis* MucR are given in Table 2.

A few years later, a gene encoding MucR was identified and characterized in another *Brucella* species, *B. abortus* [32]. The role of MucR in *B. abortus* virulence and infection of host cells was confirmed by a phenotype of a *mucR* mutant, which exhibited slow growth during in vitro cultivation and, more importantly, a virulence defect in both cultured macrophages and mice. Using a microarray analysis, a large group of genes regulated by MucR in *B. abortus* strain 2308 was identified, whose products were involved in several biological processes, including the establishment and maintenance of cell envelope integrity, polysaccharide synthesis, iron homeostasis, transcription and translation, metabolism, and signaling [32]. In total, 91 genes exhibited altered expression (>2-fold difference between the wild-type and the mutant strains), and the expression of the majority of these genes (76/91) was decreased by MucR, suggesting that this protein functions mainly as a repressor. It was confirmed that MucR binds directly to promoters of several genes (among them, *ars96 (nolR)* encoding a transcriptional regulator related to *B. abortus* virulence, *virB*, and *babR* encoding a Lux-type regulator that repressed *virB*)*,* as well as its own gene [32,33]. 

Further studies provided evidence that *B. abortus* MucR recognizes A/T-rich regions of a little sequence consensus located in the promoters of regulated genes and is a heat-stable protein (T_m_ = 63 °C) that contacts DNA mostly in the minor groove [6,22,34]. The conserved hydrophobic region at the N-terminus of this protein is responsible for the formation of its higher-order oligomer, and this oligomerization is essential for its regulatory function [6]. These features of MucR are also characteristic of another bacterial protein family named H-NS (Histone-like Nucleoid-Structuring proteins) [6,35,36,37,38]. In general, H-NS proteins are known to play important roles in nucleoid compaction. However, they also serve as gene silencers, preventing the potentially toxic expression of genes acquired by horizontal gene transfer and repressing the redundant expression of virulence genes in bacterial pathogens [39]. One of the important properties of H-NS proteins with regard to their ability to serve as gene silencers is their capacity to recognize A/T-rich DNA-target sites containing T-A steps in and around promoters. This type of protein uses these sequences as nucleation sites to form higher-order oligomers that prevent RNA polymerase (RNAP) access to these promoters [6,40]. Based on several findings for *B. abortus* MucR and *A. tumefaciens* Ros, Pirone and colleagues [6] suggested that MucR is a histone-like protein never found before in *Brucella*. Moreover, they proposed that the prokaryotic ZF proteins from the Ros/MucR family are involved in the regulation of gene expression through a mechanism similar to that used by the H-NS proteins rather than working as classical transcriptional regulators. 

Very recently, *mucR* was identified and characterized in another *Brucella* species, *B. canis* [41]. This bacterium was first isolated in 1966 from aborted tissues of beagles. *B. canis* causes reproductive failure in dogs and fever, chills, malaise, peripheral lymphadenomegaly, and splenomegaly in humans. *B. canis* MucR proved to be involved in resistance to heat stress, iron limitation, resistance to various antibiotics, and expression of flagellar genes but did not affect the growth of this bacterium (Figure 2). A lack of MucR impaired the survival of *B. canis* in macrophages and altered its virulence in mice. The *mucR* mutant was more sensitive to various antibiotics, heat stress, and iron limitation and showed increased expression of several flagellar genes (*ftcR*, *fliC*, *flgC*, *flhA*, *flgB*, *fliP*, *flgK*, and *fliF*) in relation to the wild-type strain [41]. Comparative transcriptional analysis of the *mucR* mutant vs. the wild-type strain showed significant differences in the levels of expression of 694 genes (log_2_ FC > 1 or <−1 and FDR < 0.05). In this group, 409 genes were up-regulated, and 285 genes were down-regulated in the Δ*mucR* mutant, compared to its parent strain RM6/66. As revealed by analyses of clusters of orthologous genes (COG), these genes were mainly involved in translation, ribosomal structure and biogenesis, signal transduction, energy production and conversion, intracellular trafficking, secretion, vesicular transport, and extracellular structures. Based on KEGG pathway analysis, these genes were mainly related to ribosomes, oxidative phosphorylation, protein export, aminoacyl-tRNA synthesis, and the TCA cycle. All these data confirm that *mucR* is crucial for both functioning of free-living *B. canis* cells and their virulence to hosts [41]. Similar findings were reported in 2022 for MucR of another representative of *Brucella* species, *B. ovis* [42], which is a non-zoonotic bacterium causing contagious epididymitis and other lesions in rams, which leads to significant economic losses in sheep-breeding areas. It is naturally rough due to the lack of O chains in LPS. In the *mucR* mutant, increased transcription of three genes and higher amounts of proteins encoded by these genes (i.e., *hdeA* for acid-activated chaperone HdeA, *omp25d* for an outer membrane protein Omp25d, and a gene for hypothetical protein BOV_A0299) was found. The upstream regions of these genes contained A/T-rich sequences with T-A steps, similar to the *B. abortus babR* promoter described earlier [32,33]. 

The structural homologs MucR1 and MucR2 were also identified in *Caulobacter crescentus,* a Gram-negative oligotrophic bacterium widely distributed in freshwater lakes and streams. *C. crescentus* is an important model organism for studying the regulation of the cell cycle and cellular differentiation. The MucR1 and MucR2 proteins play important roles in coordinating the expression of genes related to the cell cycle [43].

## 3. Symbiotic Bacteria 

Symbiotic nitrogen-fixing bacteria, commonly called rhizobia, possess a unique ability to establish symbiotic associations with leguminous plants and induce the formation of special organs, termed nodules, on roots and stems, wherein atmospheric nitrogen is reduced to ammonia by the enzymatic complex of nitrogenase. Rhizobia comprise a very diverse group of bacteria that belong to α- and β-*Proteobacteria* and are members of several genera, including *Sinorhizobium*, *Rhizobium*, *Mesorhizobium*, *Bradyrhizobium*, *Azorhizobium*, *Allorhizobium*, and *Methylobacterium* (α-rhizobia) as well as *Burkholderia* and *Cupriavidus* (β-rhizobia) [44]. The establishment of symbiosis is a very complex process involving an exchange of many signals of both plant and bacterial origin, with flavonoids secreted by plant roots and bacterial lipochitin oligosaccharides (Nod factors) playing key roles [45,46,47]. In addition, several cell surface components of both microsymbionts and plant hosts participate in this interaction (e.g., bacterial complex polysaccharides, including EPS, LPS, capsular polysaccharide, cyclic β-glucan (CG), flagella, and plant lectins) [48,49,50].

### 3.1. Sinorhizobium Meliloti

**Figure 3:**
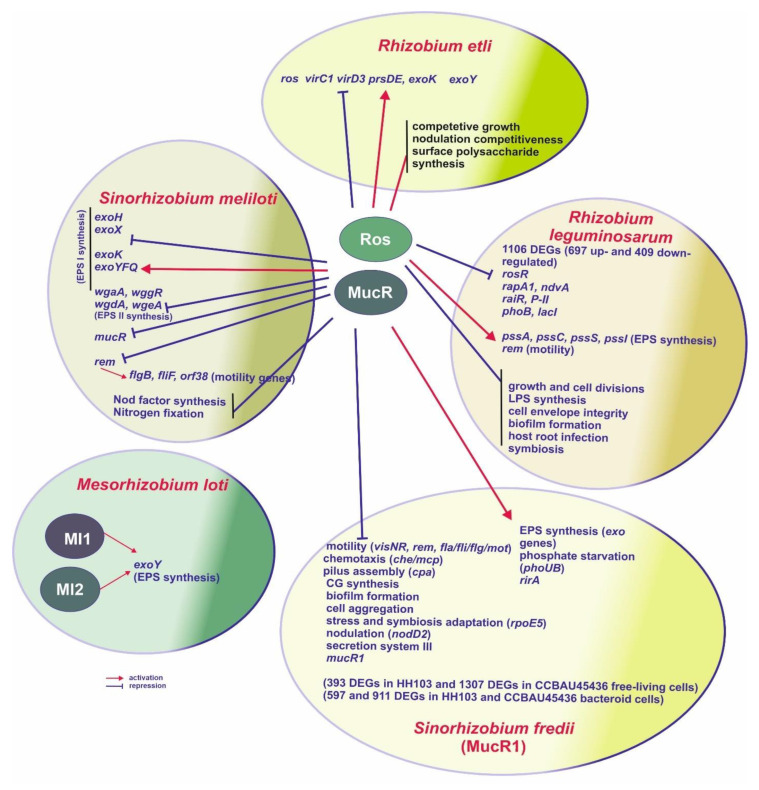
MucR/Ros proteins from different rhizobial species and their roles in regulation of expression of various genes involved in several cellular processes and adaptation to environmental stresses.

Among rhizobial bacteria, the first orthologue of *A. tumefiaciens ros* was discovered and characterized in 1995 by Pühler’s group in *Sinorhizobium meliloti* (*mucR*), a bacterium which forms nitrogen-fixing nodules on roots of plants belonging to the genera *Medicago*, *Melilotus*, and *Trigonella* [2]. This gene encodes a protein involved in regulating several cellular processes, such as motility and biosynthesis of two different kinds of EPS (EPS-I, also known as succinoglycan, and EPS-II, known as galactoglucan) required for effective symbiosis with its macrosymbiont, Nod factor synthesis, and nitrogen fixation (Figure 3, Table 2) [2,51,52,53].

A mutation in *mucR* resulted in enhanced production of EPS-II but only very low production of EPS-I. The protein encoded by this gene regulates the transcription of some *exo* genes participating in EPS-I synthesis (i.e., *exoH*, *exoX*, and *exoY*) by binding to palindromic sequences located in their promoters. The transcription of *exoH* and *exoX* was slightly increased, whereas the *exoK* and *exoYFQ* transcription slightly decreased in the *mucR* mutant. Thus, the positive role of MucR in the EPS-I synthesis was a result of both the stimulation of the expression of *exoK* and *exoYFQ genes* and the repression of *exoX* involved in the negative regulation of this process [2,51,52]. Similar to *A. tumefaciens* Ros and *B. melitensis* MucR, *S. meliloti* MucR negatively regulates its own transcription by binding to a palindromic sequence located in the *mucR* upstream region. Moreover, this protein negatively regulates the transcription of *rem,* encoding a positive regulator of motility and *wgaA*, wggR, wgdA, and wgeA genes involved in the EPS-II synthesis, working together with another regulatory protein, WggR [54,55,56,57]. This is why the inactivation of *mucR* abolishes the synthesis of EPS-I in *S. meliloti* and reduces cell motility, which, in consequence, leads to disturbances in symbiosis. MucR also stimulates the synthesis of a key symbiotic signal, the Nod factor, and represses bacterial motility and genes involved in nitrogen fixation, thus acting as an enhancer of the first steps of the nodulation process. 

### 3.2. Rhizobium Etli and Rhizobium Leguminosarum

Homologs of *A. tumefaciens* Ros, named RosR, were also identified in two species of the genus *Rhizobium*, *Rhizobium etli* (in 1997) and *Rhizobium leguminosarum* (in 2007) [3,58] (Figure 3). In *R. etli*, RosR was found to contribute to nodulation competitiveness in its host, *Phaseolus vulgaris* since a *rosR* mutant of strain CE3 was characterized by decreased competitiveness, reduced competitive growth in the rhizosphere, and a changed cell surface [59]. In 2000, Bittinger and Handelsman were the first researchers who performed a comprehensive analysis of the RosR regulon and showed that this protein regulates many genes with diverse functions in *R. etli*, including those involved in the synthesis of various polysaccharides (among them EPS), carbohydrate metabolism, and genes exhibiting sequence similarity to *virC1* and *virD3* from *A. tumefaciens* [60]. Their results indicated that RosR is involved in the expression of diverse genes in *R. etli*, some of which affect the cell surface components and nodulation competitiveness. 

In the case of *R. leguminosarum*, RosR proved to be involved in the positive regulation of EPS synthesis, similar to *S. meliloti* MucR. In this rhizobial species, three biovars: trifolii, viciae, and phaseoli were distinguished based on the type of legumes infected. Each of the biovars exhibits a narrow and specific host range: strains belonging to bv. trifolii establish symbiosis with *Trifolium* spp. plants, strains belonging to bv. viciae with *Pisum, Vicia, Lens*, and *Lathyrus* hosts, and strains belonging to bv. phaseoli with *Phaseolus* spp. plants [48,49,61,62]. Interestingly, the importance of EPS in an effective symbiosis of *R. leguminosarum* with legumes depends on the type of nodules formed by host plants. In general, EPS is essential for the symbiosis of bv. trifolii and viciae strains with their host plants, which form indeterminate-type nodules (i.e., *Trifolium*, *Pisum*, *Vicia*, *Lathyrus*, and *Lens*, belonging to the galegoid clade of the subfamily Papilionoideae), but not for bv. phaseoli strains, which establish symbiosis with *Phaseolus* spp. plants (phaseolid legumes) forming determinate-type nodules.

*R. leguminosarum rosR* is a highly conserved gene exhibiting a high similarity with *A. tumefaciens ros*, *mucR* of *S. meliloti* and *S. fredii*, and *mucR* of pathogenic *Brucella* spp., especially within their CF motifs sequences (Figure 1C). It is a single ORF located on the chromosome, and it is present in genomes of all strains belonging to the three *R. leguminosarum* biovars and a closely related species, *R. gallicum* [3,63]. This 432-bp long gene encodes a small (143-aa) protein with a mass of 15.7 kDa and, similarly to other rhizobial homologs, possesses the C_2_H_2_ ZF motif in its C-terminal domain. Studies performed by our group confirmed that *R. leguminosarum* RosR is a positive regulator of EPS synthesis, which binds to a “RosR-box” motif with a high sequence identity with the *A. tumefaciens ros*-box located in promoters of both its own gene and *pssA* encoding a glucosyltransferase engaged in the EPS synthesis [3,64,65]. Moreover, it was observed that this protein is able to form oligomeric forms since tetramers and higher forms were detected in addition to dimers [3]. To date, mechanisms involved in the regulation of the expression of genes encoding the Ros/MucR family proteins have been studied in detail only in *R. leguminosarum.* Our molecular analyses of the *rosR* upstream region indicated a very complex regulation of the transcription of this gene in *R. leguminosarum*, in which several *cis*-regulatory elements and *trans*-acting factors were engaged [3,66,67,68]. This gene possesses a very long (450 bp) upstream region, which comprises several inverted repeats of different lengths (designated as IR1 to IR6) and motifs with significant identity to consensus sequences recognized by CRP-like, PraR, PhoB, and LysR-type proteins associated with catabolic repression, quorum sensing, and phosphate- and flavonoid-dependent gene regulation, respectively [3,66,67,68]. Mutational analysis of the regulatory motifs identified in the *rosR* upstream region confirmed the significant role of some of these elements in the modulation of transcription and/or transcript stability of this gene. Our research group indicated a high level of *rosR* transcription in *R. leguminosarum* 24.2 cells, and this state was ensured by the action of two promoters, distal P1, and proximal P2, of different strengths, whose motifs are highly similar to eubacterial core elements −35 and −10, recognized by RNAP sigma factor δ^70^ [3]. *rosR* P1 functions as the main promoter, which, besides the −35 and −10 sequences, contains two additional important regulatory elements (TGN-extended −10 motif and upstream promoter (UP) element), ensuring a high level of expression of this gene [3,66]. The extended −10 motif is a 3-bp long sequence (TGG) located immediately upstream of the P1 −10 motif and is recognized by RNAP δ^70^. The UP element is a 30-bp A/T-rich sequence located upstream of the P1 −35 motif recognized by the RNAP α subunits, which facilitates the initial binding of RNAP and the subsequent steps of transcription initiation. Interestingly, these elements occur only in a small number of bacterial promoters, such as *E. coli* rRNA promoter *rrnB* P1, where this sequence resulted in an up to 30-fold effect on promoter activity [69]. To the best of our knowledge, *R. leguminosarum rosR* P1 is the first promoter with this unique structure described in rhizobia, in which both UP and TGn-extended −10 elements occur.

Further analysis confirmed that the transcription of *rosR* undergoes complex regulation. To date, the role of at least five regulatory proteins (RosR, CRP, LysR-type NodD, PhoB, and PraR) in the modulation of *rosR* expression in response to various environmental factors has been documented [67,68]. Several sequence motifs recognized by these regulatory proteins were identified in the *rosR* upstream region (RosR-box recognized by RosR, a LysR motif recognized by NodD, cAMP-CRP recognized by CRP-like protein, PHO-boxes recognized by PhoB, and PraR-box related to quorum sensing), and their function in the modulation of the *rosR* expression was confirmed. Three cAMP–CRP binding sites were found; among them, two binding sites were located upstream of P1, whereas the third binding site was located within the P2 promoter. All three motifs were functional and engaged in the transcription of this gene, indicating an influence of catabolite repression on *this process*. Moreover, the *rosR* expression increased in the presence of phosphate (0.1–20 mM) and clover flavonoids (10 μM), confirming the function of PHO-boxes (associated with phosphate regulation) and the LysR motif (associated with flavonoid-induced regulation) in the stimulation of *rosR* transcription [67,70]. Thus, several environmental factors (i.e., plant flavonoids, phosphate limitation, nitrogen starvation, and carbon source) have an influence on *rosR* transcription.

Recently, the role of a few IRs with different lengths (IR1–IR6) in the transcription of *rosR* and its mRNA stability has been elucidated in detail using a set of mutated variants of the *rosR* upstream region [67,68]. Among these motifs, IR5 was the longest and had 12-bp inverted repeats, IR2 had 11-bp long inverted repeats, whereas IR1 and IR6 had 10-bp long inverted repeats. IR1 to IR4 were located upstream (being putative targets for new yet unidentified regulatory proteins), whereas IR5 and IR6 were located downstream of two *rosR* transcriptions start sites, thus being potentially engaged in the formation of secondary structures of synthesized mRNAs [3]. IR1 and IR3 proved to exert a negative effect, whereas IR2 had a positive effect on *rosR* expression [68]. Based on an RNA decay analysis, it was confirmed that IR5 and IR6 play opposite roles in the stability of *rosR* transcripts. IR5, located at the 5′-end of both *rosR* mRNAs, played an essential role in their synthesis and protection against degradation, whereas IR6 decreased the stability of these molecules. Thus, the stem-loop structure formed upstream of the *rosR* mRNAs controlled the abundance of these molecules through transcript processing and stabilization. Usually, stem-loops stabilize transcripts when they are at the extreme 5′-end of the transcript since these double-stranded structures prevent mRNA recognition by 5′-exonuclease [71]. A comparative transcriptomic analysis of the wild-type *R. leguminosarum* Rt24.2 and the *rosR* mutant revealed the global regulatory role of RosR in rhizobial gene expression, since this protein affected the expression of genes related to various cellular processes, such as transcription and translation, signal transduction, stress adaptation, motility, synthesis of cell-surface components, as well as transport and metabolism of carbon and nitrogen sources (Figure 3) [72,73]. A majority of the large group of genes (1106) differentially expressed in the *rosR* mutant (log_2_ of mutant/wild type values > 2) were up-regulated (63%), suggesting that, like other homologs from the Ros/MucR family, RosR functions mainly as a negative regulator. Among these were *rapA1* for autoaggregation protein RapA1, *ndvA* engaged in the transport of cyclic β-glucan, and many genes encoding various transcriptional regulators (i.e., LuxR-type RaiR, nitrogen regulatory protein P-II, phosphate regulator PhoB, lactose utilization regulator LacI). On the other hand, among genes down-regulated in the *rosR* mutant were those involved in EPS synthesis (*pssA*, *pssC*, *pssS*, *pssI*) and cell motility (*rem* and a few genes encoding flagellar proteins) [74,75]. However, the RosR-box motifs identified in the promoters of RosR-regulated genes shared only low similarity with the RosR-box consensus, which may explain the necessity of the occurrence of high amounts of this protein in rhizobial cells for its regulatory function (i.e., sites with low sequence similarity to the consensus require high concentrations of regulatory proteins). According to the definition proposed by Gottesman, RosR represents global regulators on the basis of its pleiotropic phenotype and ability to regulate operons associated with different metabolic pathways [76]. These previous observations of *R. leguminosarum rosR* are in congruence with the recent findings classifying other members of the Ros/MucR family as H-NS-like proteins.

The importance of the Ros/MucR family proteins for the proper functioning of bacterial cells in both the free-living state and during symbiosis/virulence with their respective hosts is confirmed by the pleiotropic effect of a mutation in genes encoding these proteins. In the case of *R. leguminosarum*, a *rosR* mutant exhibited several biological defects, including a substantially decreased production of EPS, alterations in LPS, changed profiles of membrane and secreted proteins, changed membrane properties, and decreased motility and biofilm formation [73,77,78]. Comparative proteomic analyses confirmed the results from the transcriptomic studies and showed that many extracellular proteins related to the bacterial surface and interaction with the host plant (e.g., Ca(II)-binding cadherin-like proteins, an RTX-like protein, RapA1, and flagellins FlaA and FlaB) were substantially more abundant in the mutant than the wild-type strain. In contrast, several proteins (e.g., DppA, BraC, and SfuA) involved in the uptake of various substrates were less abundant in the mutant strain. In addition, significant differences were observed in the membrane proteins of these strains, mainly in various transport system components [73,79]. Furthermore, a *rosR* mutation led to strong disturbances in symbiosis. Although this mutant induced nodules on clover roots, they were deprived of bacteria and, in consequence, unable to fix nitrogen. RosR affected attachment and colonization of host root hairs, which are the first stages of the symbiotic process (i.e., root infection) [3,73,77,80]. 

Interestingly, multiple *rosR* copies enhance the symbiotic effectiveness and essentially increase the EPS synthesis, ensuring better adaptation of free-living *R. leguminosarum* cells to various environmental stresses, including drought, nutrient limitations, and heavy metal ions [81,82,83]. 

### 3.3. Mesorhizobium Loti

In another rhizobial species—*M. loti*, which establishes symbiosis with *Lotus japonicus*, ten genes encoding proteins with a high sequence similarity with members of the Ros/MucR family have been identified. Five of these proteins, named Ml1 to Ml5, have been recently structurally characterized [11,84,85]. It was confirmed that Ml1-Ml5 have the capacity to function as DNA-binding proteins like Ros and are able to oligomerize through their N-terminal region, likewise other members of the Ros/MucR family [84]. The DNA-binding domains of proteins Ml1, Ml2, and Ml3 require a zinc atom to fold like Ros. Interestingly, the DNA-binding domains of Ml4 and Ml5 are able to fold and bind DNA in the absence of this metal ion due to the presence of a more complex network of hydrogen bonds and a more extensive hydrophobic core than in the zinc-binding domains of proteins Ml1, Ml2, Ml3, and Ros [11,84,85]. Moreover, these domains in Ml1, Ml2, and Ml3 show a heterogeneous zinc-coordination sphere, in which aspartic acid was the second coordinating residue instead of the cysteine present in the Ros ZF domain [11,84,85,86]. Despite this structural difference, the Ml proteins share significant sequence similarity (more than 40% identity) with *A. tumefaciens* Ros [84] and have the capacity to bind to the AT-rich *vir-box*, which is a natural target site for Ros. Moreover, Ml1 and Ml2 bind to an AT-rich sequence located upstream of the *M. loti exoY* gene encoding galactosyl transferase involved in EPS synthesis in this bacterium. The core DNA-target site sufficient for DNA binding is a five-base pair AT-rich sequence (5′-AAATA-3′) containing a T-A step [11,84,86]. Interestingly, MucR from the pathogenic bacterium *B. abortus* is able to recognize Mls DNA-target sites. Genes encoding Ml1, Ml2, Ml3, and Ml5 are expressed in *M. loti* cells during their planktonic growth and in biofilms (the expression of *ml4* was not detected in these conditions) [22].

Further studies were focused on the Ml5 protein, which shows 58% sequence identity with *A. tumefaciens* Ros [85]. Interestingly, Ml5 is a zinc-lacking protein that does not contain the C_2_H_2_ motif and is nevertheless able to bind the Ros DNA target sequence with a high affinity. Baglivo and colleagues [84] demonstrated that the DNA-binding domain in Ml proteins can either use a CysAspHis_2_ coordination sphere (previously never described in DNA-binding ZF domains) or lose the structural zinc ion but still possess the DNA-binding activity. This example shows how this prokaryotic domain can overcome the metal requirement for proper folding and DNA-binding activity. All these findings indicate that the C_2_H_2_ zinc coordination sphere is generally poorly conserved within the Ros homologs, raising the question of whether the zinc ion is always preserved in these proteins. These authors demonstrated that this class of prokaryotic ZF domains is structurally very adaptable and possesses high plasticity potential. Surprisingly, single mutations do not turn off the activity but can transform a zinc-binding domain into a nonzinc-binding domain and vice versa without affecting their DNA-binding ability. In light of these findings, an evolutionary link between the prokaryotic and eukaryotic ZF domains by horizontal gene transfer from bacteria to eukaryotes seems to be very likely [84,85].

### 3.4. Sinorhizobium Fredii

*Sinorhizobium (Ensifer) fredii*, representing α-*Proteobacteria*, is closely related to *S. meliloti*, although its host range is remarkably different. *S. fredii* is able to effectively nodulate dozens of different legumes, including plants forming determinate nodules, e.g., important soybean (both American and Asiatic varieties) and cowpea crops, and plants forming indeterminate nodules (e.g., *Glycyrrhiza uralensis* and pigeon-pea) [5,45,87,88]. *S. fredii* has two genes encoding MucR, i.e., chromosomal *mucR1* and *mucR2* located on a symbiosis plasmid close to the nodulation gene *nodD1*. These genes have been characterized in detail in two *S. fredii* strains, CCBAU45436 and HH103 [5,88,89]. One of them, *mucR1*, encodes a 143–aa protein that shares a high identity with MucR and RosR from *S. meliloti* 1021 and *R. leguminosarum*, respectively (Table 2). The second copy, *mucR2*, encodes a 142-aa long protein that is 81% identical to MucR1. The MucR1 and MucR2 proteins are 100% identical in both *S. fredii* strains HH103 and CCBAU45436. Among them, MucR1 is a protein of a pleiotropic regulatory role and functional xenogeneic silencer, whereas MucR2 is unable to bind DNA due to a frameshift mutation in its C-terminal domain. As indicated for *S. fredii* strain CCBAU45436, MucR1 (but not its paralog MucR2) is essential for transcriptional activation of genes encoding conserved ion transporters required for effective nitrogen fixation of this bacterium with soybean (Figure 3) [5]. In *S. fredii* strain HH103, MucR1 also positively regulates EPS production and negatively regulates genes involved in motility, as in the case of *S. meliloti* [4]. Jiao and others [5] showed that a *mucR1* mutant of *S. fredii* CCBAU45436 forms ineffective nodules on soybean, although *nif*/*fix* genes crucial for nitrogen fixation are actively transcribed in bacteroids (bacterial forms located inside nodules). Acosta-Jurado and colleagues [4] analyzed the role of HH103 MucR1 both in the free-living state and during symbiosis with two hosts, *Glycine max* and *Lotus burttii*. Inactivation of HH103 *mucR1* led to a severe decrease in EPS synthesis and an increase in the synthesis of cyclic β-glucans, enhanced biofilm formation, and cell aggregation, and resulted in severe impairment in the symbiosis with these hosts. The pleiotropic effect of the *S. fredii mucR1* mutation was explained by the results of RNA-Seq analysis carried out for the wild-type strain and the mutant in the absence and presence of flavonoids. The numbers of differentially expressed genes (DEG) in HH103 Δ*mucR1* (defined as genes showing a fold change ≥ 3.5 or log_2_ of mutant/wild type values >1.871) were 393 and 904 in the absence and in the presence of genistein, respectively, with 265 being shared in both conditions. MucR1 was found to control the expression of hundreds of genes, among them those related to EPS synthesis, cyclic glucan transport, motility, and chemotaxis, and some genes related to the nodulation (*nod*) regulon [4]. Moreover, the MucR1 regulon within nodules exhibited significant differences from that of free-living cells [5,48,90]. In *S. fredii* CCBAU45436, the numbers of DEGs in the *mucR1* mutant in comparison to the wild-type strain (log_2_ of mutant/wild-type values >1) were 621 and 597 in free-living cells and in bacteroids isolated from soybean nodules, respectively. This protein was required for the expression of genes encoding transporters for phosphate, zinc, and elements essential for nitrogenase activity (i.e., iron, molybdenum, and sulfur) in nodules but was dispensable for regulation of *nif*/*fix* genes crucial for nitrogen fixation [5]. More recently, Li and others [91] indicated that MucR also repressed the transcription of eight genes encoding diguanylate cyclases in nodules, which are engaged in the synthesis of c-di-GMP [92]. This particle is a ubiquitous bacterial second messenger engaged in the regulation of several important cellular processes, such as biofilm formation, cell cycle and differentiation, and transition from motility to sessility [91].

Recently, Jiao and colleagues [93] have performed very interesting analyses using ChIP-seq coupled with transcriptomic data, whose purpose was establishing the function of MucR as a global xenogeneic silencer and its potential role in the adaptive regulation of foreign genes. The adaptive regulation of MucR target genes belonging to individual pangenome subsets with different conservation levels (i.e., from genus core to strain-specific genes) or replicons (i.e., chromosome, chromid, symbiotic plasmid, and two smaller accessory plasmids) was investigated in the wild-type CCBAU45436 and the *mucR* mutant in free-living and symbiotic conditions [93]. These analyses confirmed that MucR1 is a global DNA-binding protein in *S. fredii* since, in total, 1350 genes were the target genes for this regulatory protein (1307 and 911 genes were found in free-living cells and bacteroid cells, respectively, and 868 genes were shared by these two conditions) (Figure 3). Among them, MucR1 negatively regulates AT-rich core genes involved in stress and symbiosis adaptation (e.g., *rpoE5* for a sigma factor responding to general stress, *visNR*, and *rem*), genes encoding components and regulators involved in motility (*fla*/*fli*/*flg*/*mot*), chemotaxis (*che*/*mcp*), pilus assembly (*cpa*), *nodD2* encoding a negative regulator of *nod* genes, and genes encoding a type III secretion system (T3SS). In turn, MucR1 positively regulates genes related to other cellular processes, such as *exo* genes involved in EPS synthesis, *phoUB* involved in the regulation of phosphate starvation machinery, and *rirA* encoding an iron-responsive regulator involved in effective nitrogen fixation. Noteworthy, the MucR1 targets were more intensively distributed among *S. fredii* accessory genes and plasmids or genomic regions with GC% lower than the average. Moreover, the AT% of target genes was generally higher than that of non-target genes within individual replicons or core/accessory subsets of different conservation levels (ranging from genus core to strain-specific genes). High AT% is a characteristic feature of foreign genes in *Proteobacteria*; thus, the results obtained by Jiao and others in 2021 [93] indicate MucR1 as a global regulator associated with foreign genes on different replicons and across different conservation levels. These authors showed that the higher conservation status of an *S. fredii* gene was accompanied by its lower AT content and a higher transcription level. The AT content of spacer sequences between the -10 and -35 elements in MucR1 target genes was found to be significantly higher than in non-target genes. Moreover, the putative MucR1 motifs were rather degenerate and showed flexible patterns with a property of 10–11 bp periodic repeats of T or TT (TTXXXGXXXTXXXXXXXXXXTT). They were defined as the class A flexible patterns and proposed to be a putative new category of protein–DNA interaction sites. Interestingly, the high-affinity sequence bound by *A. tumefaciens* Ros (i.e., two thymines with an 11 bp interval and internal guanine—TXXXXXGXXXXXT), which is required for the interaction between Ros and DNA, shares an identical signature as the class A flexible patterns [93].

Recent studies indicate that foreign genes are characterized by the presence of high AT sequences in spacers between -35 and -10 hexamers in comparison to canonical promoters. This leads to stronger interaction of RNAP with this DNA region and, in consequence, to a higher level of transcription than in the case of genes controlled by canonical promoters [94]. What is interesting, this interaction is independent of the δ factor. 

As indicated for *S. fredii* MucR1, this regulator can extensively bind AT-rich regions across pangenome subsets of different conservation levels and replicons. MucR1 down-regulates its AT-rich target genes, which are predisposed to be transcribed at a high level due to their higher AT% in relation to non-target genes [93,95,96,97]. Jiao and colleagues [93] provided an explanation of these interesting observations and their role in the evolution of bacterial genomes and their adaptation to the changeable environment. Namely, the AT-rich signature of foreign DNA can be progressively erased during adaptive evolution, and MucR repressing the expression of these AT-rich foreign genes may be engaged in the facilitation of their integration into the rhizobial regulatory network. This adaptive regulation mechanism seems to be common for bacteria occupying various niches and having a xenogeneic silencer managing the adaptive pangenome [97,98,99,100,101,102,103,104].

In 2022, Tian’s research group proposed that the *S. fredii* MucR1 protein [105] is a novel xenogeneic silencing DNA bridger conserved in α-*Proteobacteria*. To date, no xenogeneic bridger of such regulatory function has been identified in this bacterium. The N-terminal domain of MucR1 is essential for its self-association, stability (the minimal fragment enabling self-association was 17–47 aa), and DNA-bridging ability [105]. Self-association of MucR1 was required for the formation of the DNA-MucR-DNA bridging complex and transcriptional silencing. This protein binds both minor and major grooves of DNA. Xenogeneic silencing mediated by lineage-specific DNA bridgers is known to be engaged in bacterial adaptation. These proteins, together with nucleoid-associated proteins (NAPs), are involved in the proper organization of bacterial genomes (i.e., bending and looping of DNA) [105,106,107,108]. DNA bridgers have been identified in various Gram-positive and Gram-negative microorganisms. These include H-NS (histone-like nucleoid structuring protein) in *E. coli* and *Salmonella* spp. [109], MvaT in *Pseudomonas* spp. [110,111], Lsr2 in *Mycobacterium tuberculosis* [112,113], and Rok in *Bacillus subtillis* [114,115].

Interestingly, a functional similarity between MucR1 and H-NS has been reported by Shi and others [105] since *S. fredii* MucR can be replaced in symbiosis by non-homologous H-NS from γ-proteobacterium *E. coli*. Although the N-terminal domains of proteins MucR1, H-NS, and Lsr2 show no sequence homology, similar recruitment profiles were observed across the multipartite genome of *S. fredii* (i.e., preferring AT-rich genomic islands and symbiotic plasmids with key symbiotic genes as shared targets) [105]. Moreover, the full-length protein H-NS was able to rescue the symbiotic defect of the *S. fredii mucR1* mutant. On the other hand, MucR1 functionally complemented H-S in *E. coli*. Thus, the convergently evolved MucR predisposed α-*Proteobacteria* to integrate AT-rich foreign DNA (among them symbiosis genes), whose horizontal transfer is strongly selected in the environment [105,115,116,117].

## 4. Conclusions

The Ros/MucR family is widespread in nature and encompasses numerous prokaryotic ZF-containing proteins which interact with DNA and integrate multiple biological functions, such as transcription regulation, motility, biosynthesis of surface components, biofilm formation, and competitiveness. Among members of this regulatory family are proteins from bacteria, mostly α-*Proteobacteria* colonizing various ecological niches, including symbionts and pathogens of plants and mammals. The importance of Ros/MucR proteins in the functioning of bacterial cells is confirmed by the pleiotropic effects of mutations in the genes encoding these proteins. The N-terminal domain and the ZF-bearing C-terminal region of these proteins are involved in oligomerization and DNA binding, respectively. Ros/MucR proteins possess interesting structural and functional features that represent some differences in comparison to the eukaryotic ZF-containing regulators. In the past decades, a large body of evidence showed that Ros/MucR are pleiotropic transcriptional regulators that mainly act as repressors through oligomerization and binding to AT-rich target promoters. However, more recent data on the features of Ros/MucR proteins indicate that they are convergent to those of xenogeneic silencers, such as H-NS, MvaT, and Lsr2. In light of these new facts, a novel functional model has been recently proposed for this protein family, suggesting that they act as H-NS-‘like’ gene silencers. Thus, Ros/MucR proteins have been proven to play a more global role in the evolution of bacterial genomes and their adaptation to the changeable environment. These regulators may also be engaged in the regulation of other important processes in α-*Proteobacteria*, such as replication and integration of phage-derived genes. However, these interesting aspects have not been explored until now and require further studies.

## Figures and Tables

**Table 1:** MucR/Ros homologs from different representatives of *Rhizobiaceae* family.

Protein	Length (aa)	Bacterial Species	Identity (%)	Query Cover (%)	E-Value	Accession Number
MucR	143	*Sinorhizobium meliloti* 2011	100	100	9 × 10^−87^	WP_003527383.1
MucR	143	*Sinorhizobium medicae*	99.30	100	3 × 10^−86^	WP_011974816.1
MucR1	143	*Sinorhizobium fredii* HH103	96.50	100	2 × 10^−84^	WP_014327705.1
MucR1	143	*Sinorhizobium fredii* CCBAU 45436	96.50	100	2 × 10^−84^	AWI62033
MucR	143	*Ensifer aridi*	93.01	100	1 × 10^−81^	WP_026615989.1
MucR	143	*Rhizobium herbae*	89.51	100	1 × 10^−77^	WP_220372103.1
MucR	143	*Rhizobium giardinii*	88.11	100	2 × 10^−77^	WP_018329544.1
MucR	143	*Pararhizobium* sp. YC-54	87.41	100	3 × 10^−76^	WP_264219505.1
MucR	143	*Pararhizobium polonicum*	86.71	100	1 × 10^−75^	WP_068952849.1
MucR	144	*Rhizobium herbae*	85.92	99	3 × 10^−75^	WP_209853764.1
MucR	142	*Agrobacterium rhizogenes*	85.71	97	2 × 10^−74^	WP_047463113.1
MucR	143	*Rhizobium lusitanum*	82.52	100	2 × 10^−72^	QND49923.1
MucR	143	*Rhizobium sullae*	81.82	99	1 × 10^−71^	WP_132559880.1
RosR	143	*Rhizobium leguminosarum*	81.12	99	7 × 10^−71^	AAT92553
Ros	143	*Rhizobium etli*	80.12	99	1 × 10^−71^	AAC44878
Ros	143	*Agrobacterium tumefaciens*	82.52	100	3 × 10^−74^	WP_132517515.1

**Table 2:** MucR proteins and their homology (% identity) among representatives of *Brucella* and *Mesorhizobium* genera. The MucR homologs presented here were selected on the basis of their sequence identity percentage.

Protein	Length (aa)	Bacterial Species	Identity (%)	Query Cover (%)	E-Value	Accession Number
MucR	142	*Brucella melitensis* 16M	100	100	1 × 10^−80^	WP_006266678.1
MucR	161	*Brucella ovis* ATCC 25840	99.30	100	4 × 10^−80^	ABQ60939.1
MucR	142	*Brucella abortus*	99.30	100	4 × 10^−80^	USC10419
MucR	142	*Brucella inopinata*	98.59	100	7 × 10^−80^	WP_008508511.1
MucR	142	*Brucella oryzae*	94.37	100	2 × 10^−76^	WP_104755423.1
MucR	142	*Brucella pecoris*	93.66	100	7 × 10^−77^	WP_140019901.1
MucR	143	*Paramesorhizobium deserti*	82.61	97	5 × 10^−64^	WP_068879566.1
MucR	142	*Phyllobacterium phragmitis*	80.71	98	2 × 10^−61^	WP_105740081.1
MucR	142	*Brucella endophytica*	78.87	100	6 × 10^−62^	WP_188826054.1
MucR	143	*Mesorhizobium ephedrae*	71.83	100	8 × 10^−57^	WP_106775715.1
Ml1	149	*Mesorhizobium loti*	72.14	98	2 × 10^−55^	Q989W1
Ml2	141	*Mesorhizobium loti*	71.89	98	5 × 10^−56^	Q985J6
MucR	142	*Mesorhizobium tamadayense*	72.14	98	6 × 10^−56^	WP_125005267.1
MucR	142	*Mesorhizobium* sp. ORS 3428	71.43	98	1 × 10^−55^	WP_071100028.1
MucR	141	*Mesorhizobium soli*	71.43	98	3 × 10^−55^	WP_106723787.1
MucR	141	*Mesorhizobium alhagi*	70.00	98	9 × 10^−55^	WP_008839483.1

## Abbreviations

aa	amino acids
H	histidine
C	cysteine
A	adenine
T	thymine
ZF	zinc-finger
C_2_H_2_	Cys_2_His_2_
ORF	open reading frame
IR	inverted repeats
CRP	catabolite regulatory protein
EPS	exopolysaccharide
LPS	lipopolysaccharide
CG	cyclic glucans
H-NS	Histone–like Nucleoid–Structuring
RNAP	RNA polymerase
UP	upstream promoter
COG	clusters of orthologous genes
KEGG	Kyoto Encyclopedia of Genes and Genomes
DEG	differentially expressed genes
T3SS	type three secretion system
